# Divergence in surface protein exposure between reference and clinical-derived *Candida glabrata (Nakaseomyces glabratus)* strains (CBS138 vs. BG2) – a preliminary proteomic perspective

**DOI:** 10.3389/abp.2026.16376

**Published:** 2026-07-08

**Authors:** Aneta Bednarek, Olga Barczyk-Woznicka, Justyna Karkowska-Kuleta, Elzbieta Pyza, Maria Rapala-Kozik, Dorota Satala

**Affiliations:** 1 Department of Comparative Biochemistry and Bioanalytics, Faculty of Biochemistry, Biophysics and Biotechnology, Jagiellonian University, Kraków, Poland; 2 Doctoral School of Exact and Natural Sciences, Faculty of Biochemistry, Biophysics and Biotechnology, Jagiellonian University, Kraków, Poland; 3 Department of Cell Biology and Imaging, Institute of Zoology and Biomedical Research, Jagiellonian University, Kraków, Poland

**Keywords:** biofilm, *Candida glabrata*, cell-surface shaving, cell-wall architecture, surface proteomics

## Abstract

Candida *glabrata* (currently classified as *Nakaseomyces glabratus*) is an opportunistic fungal pathogen notable for its intrinsic antifungal tolerance and ability to persist in host environments. Although strain CBS138 has served as the principal model for genetic and functional studies, accumulating evidence indicates substantial intraspecies diversity that may shape virulence, immune interactions and stress adaptation. In particular, the widely used clinical isolate BG2 differs from CBS138 in genome structure, adhesin regulation and macrophage survival, yet the extent to which these differences are reflected at the fungal cell surface remains unknown. Here, we present a comparative characterization of the surface-exposed proteomes (surfaceomes) of CBS138 and BG2 across three biologically relevant growth conditions: YPD-grown yeast-like cells, RPMI-cultured planktonic aggregates and RPMI-formed biofilms. Using trypsin shaving combined with LC–MS/MS, we identified pronounced strain- and condition-dependent differences in surface protein composition, encompassing adhesins, yapsin proteases and selected moonlighting proteins. Whereas CBS138 showed greater representation of adhesion- and interaction-related surface proteins, BG2 preferentially displayed proteins associated with cell-wall architecture and remodelling, consistent with distinct surface-mediated adaptive strategies. Transmission electron microscopy revealed condition-dependent differences in cell-wall thickness in both strains, with BG2 displaying a broader range of values and the highest thickness under biofilm conditions, providing structural context for variation in protease accessibility and surface-protein detectability. Collectively, our findings highlight substantial surfaceome plasticity in *C. glabrata* and underscore the importance of considering intraspecies diversity when interpreting host–pathogen interactions and fungal virulence pathways.

## Introduction

*Candida glabrata* (recently reclassified as *Nakaseomyces glabratus*) is an opportunistic fungal pathogen that has emerged as a major cause of candidiasis, particularly among immunocompromised individuals (Usher et al., 2023; Ciurea et al., 2020; Hassan et al., 2021; Hwang et al., 2024). Despite its close phylogenetic relationship with the non-pathogenic *Saccharomyces cerevisiae*, *C. glabrata* displays a distinct set of virulence traits, including high levels of intrinsic and acquired antifungal resistance, a compact haploid genome with notable plasticity, and the ability to persist within host cells (Ksiezopolska et al., 2021; Nickels et al., 2024). These characteristics are increasingly attributed to the microorganism’s capacity for rapid adaptation through chromosomal rearrangements, point mutations, and transcriptional rewiring, particularly in response to antifungal pressure or environmental stress (Carreté et al., 2019; Stefanini et al., 2022; Nickels et al., 2024). The medical relevance of this species is further underscored by the 2022 WHO Fungal Priority Pathogens List, which designates *C. glabrata* as a high-priority fungal pathogen requiring intensified research efforts (WHO, 2022).

Reference strains such as CBS138 (ATCC 2001) have been invaluable for establishing genetic tools and mapping fundamental virulence pathways. However, accumulating evidence demonstrates pronounced intraspecies variation, raising concerns regarding the generalization of findings derived from a single genetic background (Usher et al., 2023). Comparative studies show that CBS138 and the widely used clinical isolate BG2 differ substantially at the genomic, transcriptomic and phenotypic levels. These differences include chromosomal organization (Nickels et al., 2024), adhesin gene expression such as *EPA1* (Halliwell et al., 2012), metabolic flexibility – particularly in the utilization of tryptophan and methionine – and stress response profiles (Usher et al., 2023). Moreover, BG2 displays enhanced replication within macrophages and increased virulence in *Galleria mellonella* relative to CBS138 (Usher et al., 2023). Notably, the two strains also differ in cell wall architecture and carbohydrate exposure including mannan, β-1,3-glucan and chitin — according to electron microscopy and flow cytometry (Usher et al., 2023). Recent genetic analyses further support these structural distinctions, showing that perturbation of mannan backbone synthesis (via deletion of *MNN10*) differentially affects wall composition and immune recognition in CBS138 and BG2 (Fior Ribeiro et al., 2025).

The surface proteome, or surfaceome, constitutes the primary interface with the host environment and represents the final functional manifestation of underlying genomic and phenotypic divergence (de Groot et al., 2008; Gómez-Molero at el., 2015; López-Fuentes et al., 2018; Karkowska-Kuleta et al., 2019). Because surface-exposed proteins mediate adhesion, immune recognition, biofilm establishment and survival within phagocytic cells, even subtle differences in their abundance or accessibility may result in profoundly different infection outcomes (Cormack et al., 1999; Gómez-Molero et al., 2015; Kasper et al., 2015; Usher et al., 2023; Satala et al., 2025). Several surface proteins are dynamically regulated by environmental cues, and the differences in cell-wall architecture and the carbohydrate composition between CBS138 and BG2 directly determine which proteins are exposed on the surface and remain accessible to proteolytic shaving (Halliwell et al., 2012; Usher et al., 2023). Given that strain-dependent variation in surface protein exposure may drive distinct virulence strategies (Nickels et al., 2024), elucidating how differences in cell-wall organization and phenotypic traits shape surfaceome composition is essential for understanding host–pathogen interactions in *C. glabrata*.

To fill this knowledge gap, we conducted a preliminary comparative analysis of surface protein exposure in CBS138 and BG2 under host-relevant conditions. The resulting dataset provides a foundation for understanding how strain-specific features contribute to variation in host interaction and pathogenic potential.

## Materials and methods

### Strains and culture conditions

*C. glabrata* cells of CBS138 (ATCC® 2001™) and BG2 strains cultured 16 h, 30 °C in YPD medium (1% yeast extract, 2% soy peptone, 2% glucose, pH 6.0; Sigma, St. Louis, MO, USA) were inoculated in YPD culture media or Roswell Park Memorial Institute defined medium (RPMI 1640) (PAA Laboratories GmbH, Pasching, Austria) to obtain a model of blastospores and free-floating aggregates, respectively. The cultures were shaken at 170 rpm at 37 °C, and after 24 h or 48 h, cells were collected from the cultures for further analysis. Additionally, 1 × 10^9^ of *C. glabrata* cells from the pre-culture were inoculated into 100 mL of RPMI 1640 medium and incubated at 37 °C in sterile roller bottles (Corning Inc., New York, NY, USA) on a roller rack rotating at 3 rpm to establish a biofilm model. Similarly, cells were collected after 24 h and 48 h of incubation for further analyses.

### Transmission electron microscopy (TEM)

To examine the ultrastructure of *C. glabrata* cells under different growth conditions, transmission electron microscopy was employed. Pellet of fungal cells was fixed with 2.5% glutaraldehyde in 0.1 M cacodylic buffer overnight at 4 °C, followed by 1% osmium tetroxide for 1h at 4 °C. Before next step, samples were contrasted in uranyl acetate during 40 min. Then, they were dehydrated in graded ethanol 50%, 70%, 96% and 100%. After incubation in propylene oxide samples were embedded in Poly/Bed® 812 epoxy resin at 68 °C. In the next step, ultrathin sections, about ∼70 nm thickness were cut using microtome Leica UC7, placed on 300-mesh Formvar/Carbon grids and contrasted using uranyl acetate and lead citrate.

Imaging was done with a JEOL JEM 2100HT (Jeol Ltd, Tokyo, Japan) transmission electron microscope TEM that was used at an accelerating voltage of 80 kV. Images were taken by using 4 k × 4 k camera (TVIPS) equipped with EMMENU software ver. 4.0.9.87.

### Digestion of cell wall proteins with trypsin

*C. glabrata* cells collected after 24 h and 48 h of incubation in appropriate media were washed 3 times with PBS solution via a series of 5 min, 3,000 × g centrifugations. Cell concentration was estimated by measuring optical density at 600 nm, after which 5 × 10^8^ cells each were transferred to tubes and washed 3 times in 25 mM ammonium carbonate solution under the same parameters. The cells were then resuspended in 100 µL of 25 mM ammonium carbonate with 5 mM dithiothreitol (DTT) followed by the addition of 10 µL of trypsin (Promega, Madison, WI, USA), gently mixed and incubated for 5 min at 37 °C. After the time, the cells were centrifuged at 3,500 × g, 5 min, and the supernatants were transferred and filtered through a filter with a pore diameter of 0.22 µm (Merck, Darmstadt, Germany) into new tubes and left at 37 °C overnight. The next day, trifluoroacetic acid (TFA) (Sigma) was added to the samples to a final concentration of 0.1%, incubated at 4 °C for 15 min, then centrifuged at 12,000 × g, 15 min. The supernatant was transferred to low-binding tubes (Neptune Scientific, Sunnyvale, CA, USA) and dried using a SpeedVac (Martin Christ, Osterode am Harz, Germany). All conditions and time points were analysed in three independent biological replicates. To ensure full comparability between strains and culture conditions, identical cell numbers (5 × 10^8^ cells per sample), trypsin concentration and digestion times were used for all preparations. These parameters were standardized to minimize technical variation in peptide release during the shaving procedure. To verify that shaving conditions did not compromise cell integrity, the membrane integrity of the remaining cells was assessed using SYTOX® Green (Invitrogen Life Technologies, Carlsbad, CA, USA) and Trypan Blue staining (Sigma).

### Analysis by mass spectrometry

The mass spectrometry analysis was performed as described previously (Karkowska-Kuleta et al., 2019). In brief, the peptide precipitate was resuspended in 110 µL Loading Buffer (10% acetonitrile, 0.1% formic acid), shaken for 5 min at 110 rpm and sonicated in an ultrasonic bath another 5 min. The sample was then centrifuged to remove precipitated proteins and the supernatant was transferred to glass vials (Polygen, Gliwice, Poland). Samples were analysed on an HCT Ultra mass spectrometry instrument (Bruker, Bremen, Germany) with an ETDII ion trap equipped with an electrospray ion source (ESI) coupled to a high-performance liquid chromatography (Dionex Ultimate 3000) system. The separation was carried out on a 100 mm × 2.1 mm Aeris 3.6 μm PEPTIDE XB-C18 column (Phenomenex, Torrance, CA, USA) in a 10%–55% gradient of 0.1% formic acid in 80% acetonitrile for 60 min with a flow rate of 0.2 mL/min. After separation, the peptides were analysed in standard MS/MS mode with simultaneous fragmentation of the most intense precursor ions using collision ion dissociation (CID) and electron transfer dissociation (ETD). The raw data obtained from the instrument were pre-processed using Data Analysis 4.0 software (Bruker), generating files in Mascot Generic Format. The peak lists were searched using the local Mascot server (v.2.3.0; Matrix Science, London, UK) against a *Candida* (*Nakaseomyces*) protein database downloaded from NCBI, with the appropriate taxonomic restriction, and with automatic target–decoy searching enabled. Search parameters included: enzyme specificity - trypsin; up to two missed cleavages; variable modification - methionine oxidation (M); C^13^ number – 1; mass values - monoisotopic; peptide mass value tolerance - ±0.3 Da; fragment ion mass tolerance - ±0.3 Da; charge states - 1+, 2+, 3+. The experiment was performed in three biological replicates. Protein identification confidence was assessed using Mascot-reported target–decoy-based false discovery rate (FDR) estimates at the level of individual LC–MS/MS runs, and only those with FDR values not exceeding 2.5% were retained for further analysis. To ensure a conservative comparative dataset, only proteins supported by at least two distinct peptides and detected in at least two out of three biological replicates were included in the comparative quantitative analysis. The relative abundance of proteins was determined using the spectral abundance factor (SAF) (McIlwain et al., 2012), calculated according to the formula:

$$\text{SAF}=\left(\text{SpC}\;/\;L\right)$$

where SpC is the number of spectra assigned to a protein and L is its length in amino acids. The mass spectrometry proteomics data have been deposited to the ProteomeXchange Consortium via the PRIDE partner repository (Perez-Riverol et al., 2022). Graphical data presentation and functional enrichment analyses were performed using the FunRich software (Fonseka et al., 2021; Pathan et al., 2015; Pathan et al., 2017).

## Results

### Identification of surface-exposed proteins across growth conditions

To investigate strain-specific differences in surface protein exposure, *C. glabrata* strains CBS138 (ATCC 2001) and BG2 were cultured under three distinct growth conditions representing different morphological states: as budding blastospores in rich YPD medium, as free-floating cellular aggregates in RPMI 1640 medium (Erlenmeyer flask cultures), and as mature biofilms in dynamic RPMI cultures using the roller-bottle model. In each condition, cells were harvested at two time points (24 h and 48 h), and surface-exposed proteins were selectively digested using the trypsin-shaving method (Karkowska-Kuleta et al., 2019). The resulting peptides were analysed with LC-MS/MS, and protein abundance was estimated semi-quantitatively using the spectral abundance factor (SAF), which accounts for both spectral counts and protein length (Zybailov et al., 2006). Across all tested conditions, surface-exposed proteins were successfully identified in each of the six growth models for both CBS138 and BG2 strains. In CBS138, 54 and 65 proteins were detected after 24 h and 48 h of growth in YPD medium, respectively. Under RPMI planktonic conditions, the numbers were lower, with 13 proteins identified at 24 h and 9 at 48 h. In the biofilm model using the roller-bottle system, 30 and 22 proteins were detected after 24 h and 48 h, respectively. In contrast, the clinical isolate BG2 showed a markedly different profile. In YPD cultures, only 9 and 12 proteins were identified at 24 h and 48 h, respectively. In RPMI planktonic conditions, the number of identified proteins remained within a similar range, with 13 proteins at 24 h and 9 at 48 h. The surfaceomes of BG2 biofilms included 11 proteins at 24 h and 21 at 48 h. These data confirm substantial variation in surfaceome composition across time points, culture models, and genetic backgrounds. Overall, CBS138 exposed more detectable proteins in nutrient-rich YPD, whereas BG2 tended to show relatively greater detectability under selected RPMI-based conditions.

### Comparison of strain-specific protein sets (Venn diagrams)

**FIGURE 1:**
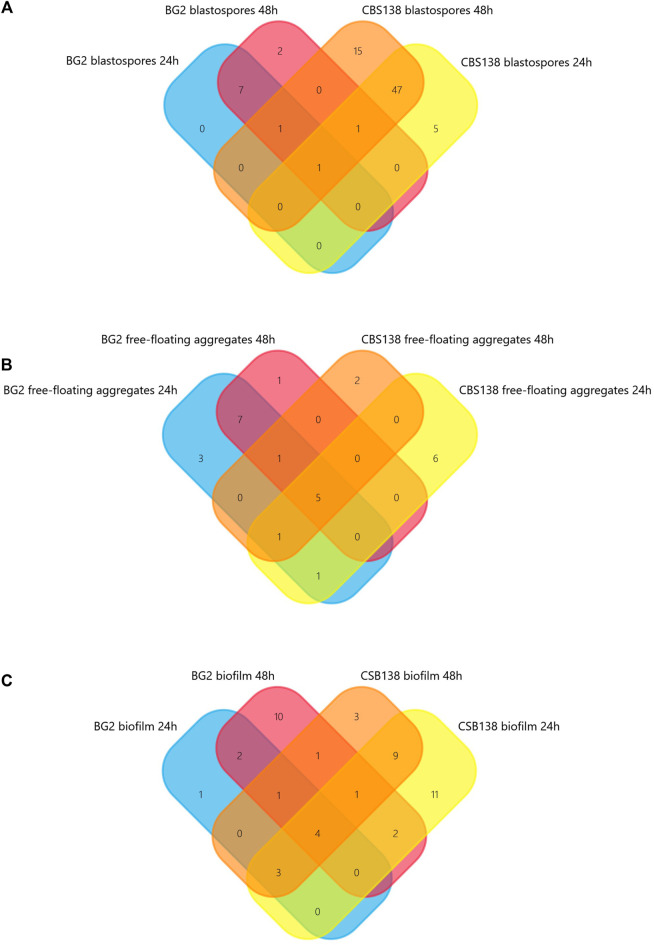
Venn diagrams comparing the sets of surface-exposed proteins identified in *C. glabrata* CBS138 and BG2 strains under three different culture conditions: **(A)** YPD medium, **(B)** RPMI planktonic growth (free-floating aggregates), and **(C)** RPMI biofilm model (roller bottle). Each diagram displays the number of proteins unique to each strain and shared between strains after 24 h and 48 h of incubation (combined). The limited overlap between strains across all conditions reflects substantial differences in surfaceome composition and strain-specific adaptation to the environment.

To compare strain-specific surfaceome profiles across distinct growth forms, three Venn diagrams were generated using FunRich software (Fonseka et al., 2021; Pathan et al., 2015; Pathan et al., 2017), comparing the sets of proteins identified in CBS138 and BG2 under each culture model: YPD-grown blastospores, RPMI planktonic (free-floating) cells, and RPMI biofilms (Figure 1). The overlap between the two strains was limited in all conditions, with both strains exposing condition-dependent sets of detectable proteins. Notably, the highest degree of overlap between strains was observed under RPMI planktonic conditions, where several proteins were consistently detected in both CBS138 and BG2. In contrast, biofilm and YPD conditions showed more pronounced strain-specific differences in the detected surface protein sets. To place these overlaps in a quantitative context, we compared the total number of proteins identified per strain, condition and time point, which revealed strongly model-dependent differences between CBS138 and BG2. Because surface shaving captures only protease-accessible regions on intact cells, identification counts primarily reflect surface accessibility rather than total proteome coverage. All conditions and time points were analysed in three independent biological replicates, supporting the robustness of the observed strain- and model-dependent patterns. In YPD, CBS138 yielded substantially more identifications than BG2 at both 24 h (54 vs. 9) and 48 h (65 vs. 12), whereas under RPMI planktonic conditions BG2 yielded only slightly more identifications than CBS138 at 24 h (18 vs. 13) and 48 h (14 vs. 9). In the biofilm model, CBS138 produced more identifications at 24 h (30 vs. 11), with a smaller difference at 48 h (22 vs. 21), indicating partial convergence in detection during biofilm maturation. Overall, these comparisons highlight strong, condition-dependent divergence in surface exposure between strains, while also indicating that overlap between strains was greatest under RPMI planktonic conditions.

### Ultrastructural analysis of the cell wall by TEM

**FIGURE 2:**
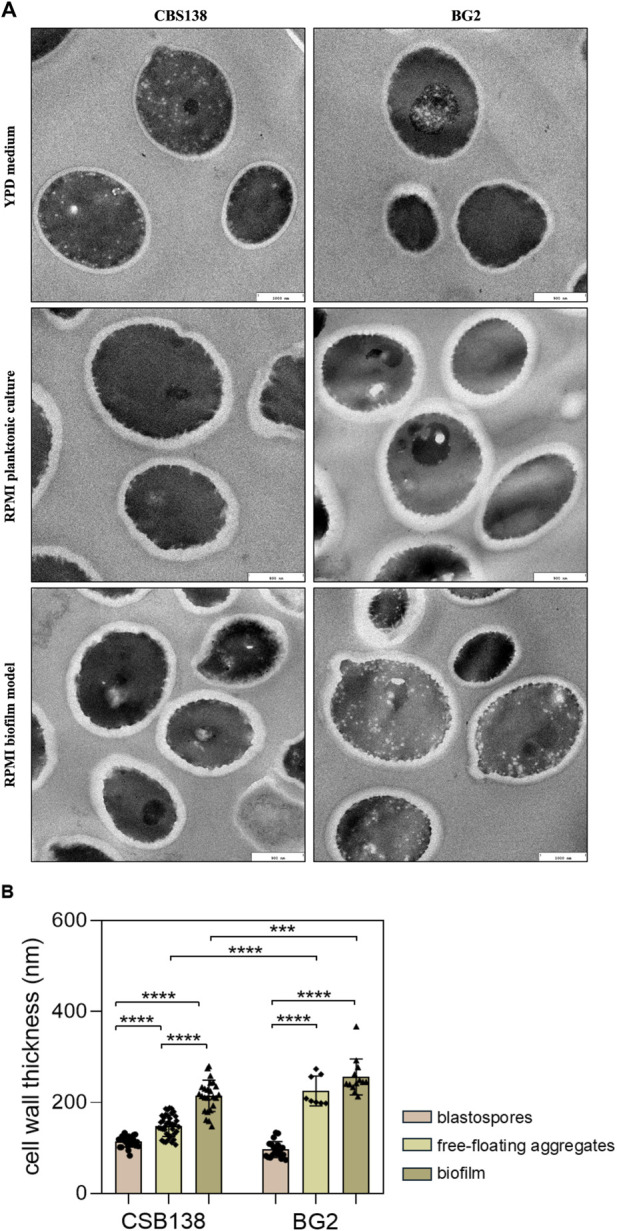
Transmission electron microscopy of *C. glabrata* CBS138 and BG2. **(A)** Representative TEM images of CBS138 (left) and BG2 (right) after 24 h of growth under three culture conditions: YPD medium, RPMI planktonic culture and RPMI biofilm model. Images illustrate overall cell-wall morphology using negative staining with uranyl acetate. Scale bars: 900 nm or 1,000 nm, as indicated. **(B)** Quantification of total cell-wall thickness measured across multiple fields and biological replicates for each strain and growth condition. Individual measurements are shown together with mean ± SD. Statistical significance was assessed by two-way ANOVA (factors: strain and growth condition) followed by Šídák’s multiple-comparisons test to compare growth conditions within each strain. Significance is indicated as ***p < 0.001,****p < 0.0001.

Transmission electron microscopy (TEM) was used to examine whether strain-specific differences in surface protein detection might be associated with variation in cell-wall architecture. For both CBS138 and BG2, representative TEM images were acquired after 24 h of growth under the same three culture conditions used for surface-shaving assays – YPD-grown blastospores, RPMI free-floating cells and RPMI biofilms – and total cell-wall thickness was quantified by taking multiple independent measurements per cell (at distinct positions along the cell perimeter) for all analysable cells within each field of view; cell-level values were then pooled within each biological replicate to obtain replicate-level distributions. (Figure 2, Supplementary Figure S1, Supplementary Table S1). Across all conditions, measurable differences in total cell-wall thickness were observed between growth models. In YPD-grown cultures, CBS138 showed only a marginally thicker wall than BG2, and the two strains largely overlapped in their measurements. In contrast, growth in RPMI planktonic (free-floating) conditions was associated with a clear increase in wall thickness relative to YPD for both strains, with the most pronounced shift observed for BG2.

To quantify these observations, we performed a two-way ANOVA with “strain” and “growth condition” as fixed factors. The analysis identified significant main effects of growth condition and strain, as well as a significant strain × condition interaction (condition: F(2,129) = 265.9, p < 0.0001; strain: F(1,129) = 43.66, p < 0.0001; interaction: F(2,129) = 31.78, p < 0.0001). In practical terms, this means that (i) wall thickness differs strongly between growth models (YPD < RPMI planktonic < biofilm), (ii) CBS138 and BG2 differ in overall thickness, and (iii) the magnitude of the RPMI-associated thickening is not the same for both strains—consistent with the pronounced YPD-to-RPMI increase observed in BG2. These ultrastructural measurements provide a structural framework for interpreting strain- and condition-dependent differences in protease accessibility observed in the surface-shaving experiments.

### Differential abundance of surface proteins (SAF analysis)

**FIGURE 3:**
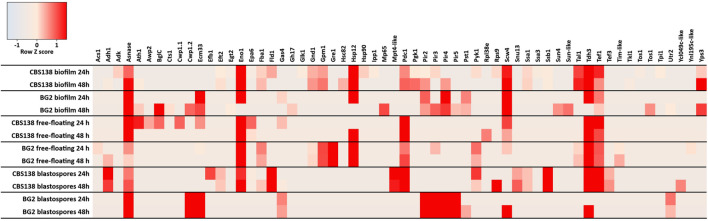
Heatmap showing the relative abundance of selected surface-exposed proteins identified in *C. glabrata* CBS138 and BG2 strains across six culture conditions: YPD medium (24 h and 48 h), RPMI planktonic culture (24 h and 48 h), and RPMI biofilm model (24 h and 48 h). Abundance values were calculated using the spectral abundance factor (SAF). Values were scaled as row-normalized Z-scores, allowing comparison of condition-dependent abundance patterns within each protein regardless of absolute intensity differences.

SAF-based analysis revealed distinct abundance profiles depending on the strain, medium, and time point (Supplementary Table S2 in Supplementary Materials). In CBS138, RPMI planktonic cultures were characterized by the presence of canonical adhesins, including Epa6 and Awp2, whereas RPMI biofilm conditions showed stronger representation of surface-associated proteins involved in host interaction and cell wall organization, including Yps3 and Scw4. By contrast, BG2 biofilm cultures, particularly at 48 h, showed increased representation of proteins linked more broadly to cell wall architecture and remodelling, such as Ecm33, Cwp1.2 and Pir4. Moreover, several metabolic enzymes previously described as moonlighting proteins – such as enolase (Eno1), glyceraldehyde-3-phosphate dehydrogenase (Tdh3), fructose-bisphosphate aldolase (Fba1), and phosphoglycerate kinase (Pgk1) – were detected across multiple conditions, with variable relative abundance depending on the strain and growth model. These findings indicate substantial variation in abundance of surface proteins, including moonlighting proteins, under conditions mimicking host environments (primarily RPMI-based models). To facilitate comparison of relative abundance patterns across growth conditions, Figure 3 presents row-normalized (Z-score) values. To complement this descriptive overview, SAF values for four proteins detected across multiple growth conditions – two classical cell wall proteins (Scw4 and Pir4) and two moonlighting proteins (Eno1 and Tdh3) – were additionally compared using distribution plots and exploratory statistical analysis (Supplementary Figure S2 and Supplementary Table S3 in Supplementary Materials).

### Functional classification of surface-exposed proteins (GO analysis)

**FIGURE 4:**
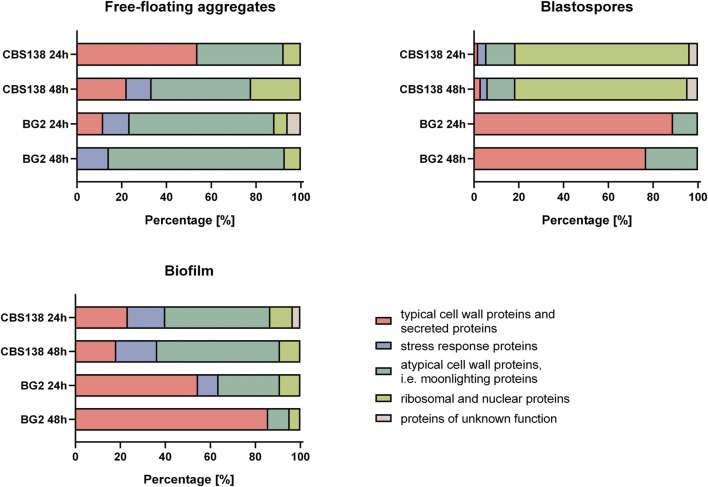
Functional classification of surface-exposed proteins identified in *Candida glabrata* CBS138 and BG2 strains based on Gene Ontology annotations retrieved from the *Candida* Genome Database. The analysis was performed using FunRich software. Proteins were categorized according to their predicted biological roles across six culture conditions: YPD (24 h and 48 h), RPMI planktonic (24 h and 48 h), and RPMI biofilm (24 h and 48 h).

Functional classification of the surface-exposed proteins revealed marked strain- and condition-dependent differences in the distribution of functional categories (Figure 4). In YPD-grown cells, CBS138 displayed a higher proportion of ribosomal, nuclear and metabolic moonlighting proteins, whereas BG2 was enriched in classical cell wall and secreted proteins such as Cwp1, Scw4 and Pir family members. Under RPMI planktonic conditions, the surfaceome profiles differed depending on both strain and growth form. In free-floating aggregates, both strains showed a substantial contribution of moonlighting proteins (e.g., Tdh3, Eno1), with this feature being particularly evident in BG2. In the biofilm model, CBS138 displayed a more mixed surfaceome composition, with contributions from both wall-associated and moonlighting proteins. In contrast, BG2 biofilms, particularly at 48 h, were enriched in remodelling and cell wall integrity factors–including Cwp1.2, Ecm33, Pir3 and Pir5 – with reduced detection of adhesins. Interestingly, Yps-family proteases were detected in the biofilm condition – Yps3 was present in CBS138 biofilms at both time points and in BG2 biofilm at 48 h. Together, these observations highlight clear differences in surfaceome functional composition between strains and across growth conditions.

## Discussion

Understanding how *C. glabrata* adapts its cell surface to different environments is essential, as surface-exposed proteins directly influence adhesion, immune recognition and antifungal resilience. However, most studies rely on a single reference strain, despite growing evidence of substantial intraspecies diversity (Usher et al., 2023). The present work addresses this gap by initially comparing the surfaceomes of CBS138 and BG2 under multiple growth conditions and by linking these proteomic profiles to differences in cell wall architecture. Together, these complementary approaches allow a more integrated view of how strain-specific and environment-dependent factors shape the accessible surface landscape of *C. glabrata.*

To capture temporal changes in biofilm-associated surface remodelling, shaving was performed at 24 h and 48 h, representing early and mature stages of *Candida* biofilm development in standard models (Rodrigues et al., 2017; Kucharíková et al., 2011; Seneviratne et al., 2009). Within this framework, proteomics-based surface shaving provides direct evidence of surface accessibility by identifying peptides derived from protease-accessible regions on intact cells (Olaya-Abril et al., 2014; Pauwels et al., 2022), which is particularly informative for host–pathogen interactions and environmental adaptation (Arana et al., 2009; Aggarwal et al., 2023).

The utility of shaving-based proteomics is especially evident in fungal pathogens, whose cell walls represent highly dynamic and structurally complex interfaces. In *Candida* species, the cell wall consists of an inner scaffold of chitin cross-linked with β-1,3- and β-1,6-glucans and an outer layer enriched in mannoproteins, within which a diverse repertoire of surface-associated proteins is embedded (Satala et al., 2020). These proteins differ in their modes of attachment, including glycosylphosphatidylinositol (GPI) anchors, alkali-labile Pir linkages and non-covalent interactions with the polysaccharide matrix. In addition to classical cell wall proteins, the fungal surface frequently displays moonlighting proteins – cytosolic enzymes or chaperones that acquire adhesive, enzymatic or immune-modulatory functions upon externalization (Gil-Bona et al., 2018; Satala et al., 2020). Early large-scale shaving studies in *C. albicans* established the breadth and context-dependence of fungal surface exposure across growth forms (Vialás et al., 2012; Gil-Bona et al., 2015; Gil-Bona et al., 2018). In *C. glabrata* CBS138, niche-mimicking conditions yield distinct surfaceomes enriched in dynamically exposed moonlighting proteins (Karkowska-Kuleta et al., 2019), and independent infection-related models further support that cell wall remodelling can modulate adhesin accessibility and host interactions, including regulation of Epa-family exposure (Zajac et al., 2016; Zhang et al., 2024; Shinohara et al., 2025). Although shaving-based proteomics is highly informative for defining the protease-accessible surfaceome, it does not by itself provide definitive proof of cell wall localization for each identified protein. This is particularly important in the case of newly detected atypical or moonlighting proteins. Accordingly, the proteins identified here are interpreted as surface-accessible under the tested conditions, whereas their exact mode of association with the cell wall should be verified by complementary orthogonal approaches performed on intact cells, such as immunofluorescence-based localization on non-permeabilized cells, protease accessibility or protease protection assays, analysis of surface-enriched fractions by immunoblotting, or surface-labeling strategies including cell-surface biotinylation followed by affinity capture (Satala et al., 2026; Bonn et al., 2018; Besingi and Clark, 2015). At the same time, the surface accessibility of this class of proteins has been supported by our previous studies on selected candidal moonlighting proteins using antibody-based detection on intact cells, including fluorescence microscopy, flow cytometry, microplate-based immunodetection, and immunoblotting of cell-wall-associated fractions, as detailed for Eno1 (Karkowska-Kuleta et al., 2021), Tdh3 (GAPDH) (Bednarek et al., 2024), and Tpi1 (Satala et al., 2021).

Interestingly, in our study, the total number of detectable surface proteins was lower in BG2 than in CBS138 under several growth conditions, most notably during cultivation in YPD. To determine whether this strain-dependent disparity in surface protein detectability has a structural basis, we next examined cell wall architecture using transmission electron microscopy. Ultrastructural analysis revealed substantially thicker cell walls in BG2 under RPMI planktonic conditions, whereas no significant differences in total wall thickness were observed between the strains while growing in YPD. At first glance, this appears counterintuitive given the pronounced discrepancy in surfaceome size in YPD-grown cells, where 54 proteins were identified in CBS138 but only 9 in BG2. However, this apparent inconsistency can be resolved when cell wall accessibility is considered independently of overall wall thickness. As shown by Usher et al. (2023) CBS138 is characterized by a more open and highly fibrillar outer mannan layer, with long, loosely organized fibrils and increased exposure of mannan, β-1,3-glucan and chitin. In contrast, BG2 displays a more compact and densely packed outer layer, accompanied by 2- to 4-fold lower fluorescence signals for these carbohydrate components. Consistent with this model, Fior Ribeiro et al. (2025) showed that disrupting Mnn10 reduces inner/outer wall thickness yet markedly increases β-glucan, mannan and chitin exposure in both BG2 and CBS138, reinforcing that wall “tightness”, rather than thickness, governs molecular accessibility and protease penetration. TEM thickness measurements capture size but not porosity, fibrillar organization or chemical accessibility – key determinants of trypsin-based shaving efficiency.

Accordingly, the relatively thick but porous outer wall of CBS138 may remain readily permeable to proteolytic enzymes, allowing efficient release of peptides derived from GPI-anchored proteins and embedded moonlighting factors. Conversely, the compact and tightly cross-linked mannan network of BG2 likely constitutes a physical barrier that restricts protease penetration, limiting protein detectability to a subset of the most exposed components, including Pir proteins or Ecm33. This interpretation is further supported by the observation that BG2 exhibits very low protein detectability in YPD despite a wall thickness comparable to that of CBS138, indicating that surface accessibility – rather than wall size *per se* – governs proteolytic release.

Notably, BG2 showed greater surface proteins detectability under selected RPMI-based conditions, coinciding with an increase in wall thickness. This suggests that host-mimicking environments can induce substantial remodelling of the BG2 cell wall matrix, leading to altered permeability and surface exposure. Together with the strain- and condition-dependent profiles observed here, this supports a multifactorial view of *C. glabrata* wall remodelling driven by enzymatic mannan processing, environmental cues and genetic background, which collectively fine-tune surface accessibility and virulence potential (Fior Ribeiro et al., 2025). Such dynamic behaviour is consistent with previous reports demonstrating strong activation of cell wall integrity pathways in BG2 and its pronounced capacity to adapt wall architecture in response to host-associated stresses.

Several of the surface proteins identified in this study are of particular interest due to their well-established roles in virulence-associated processes. The first group comprises members of the Epa family, which are GPI-anchored adhesins involved in epithelial adherence and tissue colonization (Halliwell et al., 2012; Usher et al., 2023). Adhesin expression in *C. glabrata* is known to be tightly regulated by subtelomeric silencing mechanisms mediated by Sir3. In particular, *EPA1* expression has been shown to be highly heterogeneous in BG2 as a consequence of weakened Sir3-dependent repression, whereas CBS138 exhibits a more uniform adhesin expression profile (Halliwell et al., 2012; Nickels et al., 2024). These regulatory differences provide a plausible molecular basis for several strain-specific patterns observed in our data, including the stronger representation of adhesins in CBS138 surfaceomes and the more condition-dependent adhesin exposure in BG2. Finally, Mp65 was detected under selected strain–condition combinations, consistent with its established roles in wall integrity, adhesion/biofilm formation and immunogenicity (Sandini et al., 2011).

A second group of proteins identified in this study comprises moonlighting proteins whose surface exposure confers additional adhesive or immune-modulatory functions. In *C. glabrata*, Tdh3 has been shown to bind vitronectin and plasminogen, with its anchoring to the cell wall mediated by the adhesin Epa6, linking “moonlighting” protein exposure directly to adhesin-dependent surface organization (Bednarek et al., 2024). Similarly, Tpi1 interacts with human extracellular matrix proteins, and antibody-based analyses demonstrated a significantly higher surface abundance of Tpi1 following growth in RPMI medium, indicating environmentally regulated externalization (Satala et al., 2021). For other moonlighting proteins detected here, direct functional studies in *C. glabrata* are limited. However, data from *C. albicans* show that several surface-exposed metabolic enzymes and chaperones, including Eno1, Pgk1, Fba1 and Tdh3 (among others), are immunoreactive during invasive candidiasis (Pitarch et al., 2006; Pitarch et al., 2011). Notably, antibody responses to these proteins are functionally diverse, ranging from protective effects, such as antibodies against Cdc19 or Fba1, to associations with adverse outcomes, as reported for anti-Tdh3 or anti-Ssb1 antibodies, highlighting the complex immunological consequences of moonlighting protein exposure (Medrano-Díaz et al., 2018).

In addition, several surface-exposed aspartyl proteases of the Yps family, including Yps3, were identified in our analyses. Previous studies have primarily linked Yps proteases to the maintenance of cellular homeostasis, cell wall integrity and general stress adaptation (Patra and Kaur, 2024; Rasheed et al., 2018; Askari et al., 2022). However, more recent work has expanded this view by demonstrating that selected Yps proteases are capable of degrading host-derived antimicrobial peptides, including LL-37, histatin-5 and the kininogen-derived peptide NAT26 (Satala et al., 2025). Beyond direct peptide neutralization, Yps have also been shown to interfere with epithelial–immune cell communication by impairing IL-8 secretion from epithelial cells, thereby limiting neutrophil recruitment and reducing neutrophil-mediated killing of *C. glabrata* (Patra and Kaur, 2024). Together, these findings suggest that Yps proteins may contribute directly to immune evasion at multiple levels, providing new insight into their role in *C. glabrata* pathogenesis.

While individual proteins offer mechanistic clues, examining functional trends across the surfaceome provides a broader perspective on strain-specific adaptation. In our study, under RPMI planktonic conditions, CBS138 was associated with the appearance of canonical adhesins such as Epa6 and Awp2, whereas BG2 showed a profile marked by a strong contribution of moonlighting proteins alongside selected wall-associated factors. During biofilm formation CBS138 retained a mixed surfaceome composition, while BG2 showed a more pronounced representation of proteins associated with cell wall architecture and remodelling, including Cwp1.2, Ecm33, Mp65 and Pir family proteins. Together, these observations support the view that CBS138 and BG2 adopt distinct surfaceome-level responses to host-like environments, with CBS138 maintaining a broader multifunctional profile and BG2 shifting more strongly toward wall remodelling and reinforcement. These mechanistic differences align with previously reported phenotypic traits–including the enhanced macrophage replication and increased virulence of BG2 (Usher et al., 2023) – and emphasise that intraspecies variation in *C. glabrata* can translate into markedly different surface-mediated pathogenic strategies. In line with this, beyond wall architecture itself, Fior Ribeiro et al. (2025) also reported strain-dependent immunological consequences of *MNN10* deletion: the BG2 *mnn10Δ* mutant displayed reduced uptake and intracellular survival in macrophages yet triggered stronger GM-CSF secretion, whereas the CBS138 *mnn10Δ* mutant more strongly activated the EphA2 receptor, reflecting enhanced β-glucan exposure. These immunological differences highlight that wall remodelling and glycan masking do not simply affect surface accessibility but profoundly shape host recognition and inflammatory signalling. Taken together, these findings reinforce the concept that CBS138 and BG2 differ not only in their structural and proteomic surface landscapes but also in the way these architectures are sensed by the immune system, ultimately dictating strain-specific trajectories of virulence and host adaptation. Collectively, these strain-specific differences in wall remodelling and glycan masking may also contribute to divergent *in vivo* tissue tropism and colonization patterns, by shaping niche-dependent adhesion, immune sensing, and persistence across anatomical sites. Such findings underscore the need to consider strain-specific surfaceome architecture when interpreting host–pathogen interactions and designing therapeutics targeting the fungal cell surface.

In conclusion, our findings show that *C. glabrata* surfaceome composition is highly plastic and strongly dependent on both strain identity and growth environment. The parallel variation in cell wall thickness supports a structural basis for differences in protease accessibility and surface protein detection. By extending surfaceome analysis beyond standard YPD conditions and incorporating ultrastructural evidence, this study highlights the critical impact of intraspecies diversity on surface architecture and underscores the necessity of considering strain-specific traits when interpreting virulence- or immunity-related phenotypes.

## Data Availability

The datasets generated and analysed during the current study are available in the Cracow Open Research Data Repository, https://doi.org/10.57903/UJ/7MTM4L. The mass spectrometry proteomics data have been deposited to the ProteomeXchange Consortium via the PRIDE partner repository with the dataset identifier PXD074144.
