# Supplementary material for: Divergence in surface protein exposure between reference and clinical-derived Candida glabrata (Nakaseomyces glabratus) strains (CBS138 vs. BG2) – a preliminary proteomic perspective
Source: Acta Biochim Pol. 2026 Jul 8;73:16376. doi: 10.3389/abp.2026.16376 (PMC13388231; doi:10.3389/abp.2026.16376)
Supplement: Supplementary file 1 [file DataSheet1.pdf]

SUPPLEMENTARY DATA

Divergence in surface protein exposure between reference and clinical-derived *Candida glabrata (Nakaseomyces glabratus)* strains (CBS138 vs. BG2) – a preliminary proteomic perspective

Aneta Bednarek[1,2], Olga Barczyk-Woznicka[3], Justyna Karkowska-Kuleta[1], Elzbieta Pyza[3], Maria Rapala-Kozik[1], Dorota Satala[1*]

[1]Department of Comparative Biochemistry and Bioanalytics, Faculty of Biochemistry, Biophysics and Biotechnology, Jagiellonian University, Gronostajowa 7, 30-387 Kraków, Poland

[2]Doctoral School of Exact and Natural Sciences, Faculty of Biochemistry, Biophysics and Biotechnology, Jagiellonian University, Gronostajowa 7, 30-387 Kraków, Poland.

[3]Department of Cell Biology and Imaging, Institute of Zoology and Biomedical Research, Jagiellonian University, Gronostajowa 9, 30-387 Kraków, Poland

* Correspondence: phone number: 48126646524; e-mail: dorota.satala@uj.edu.pl

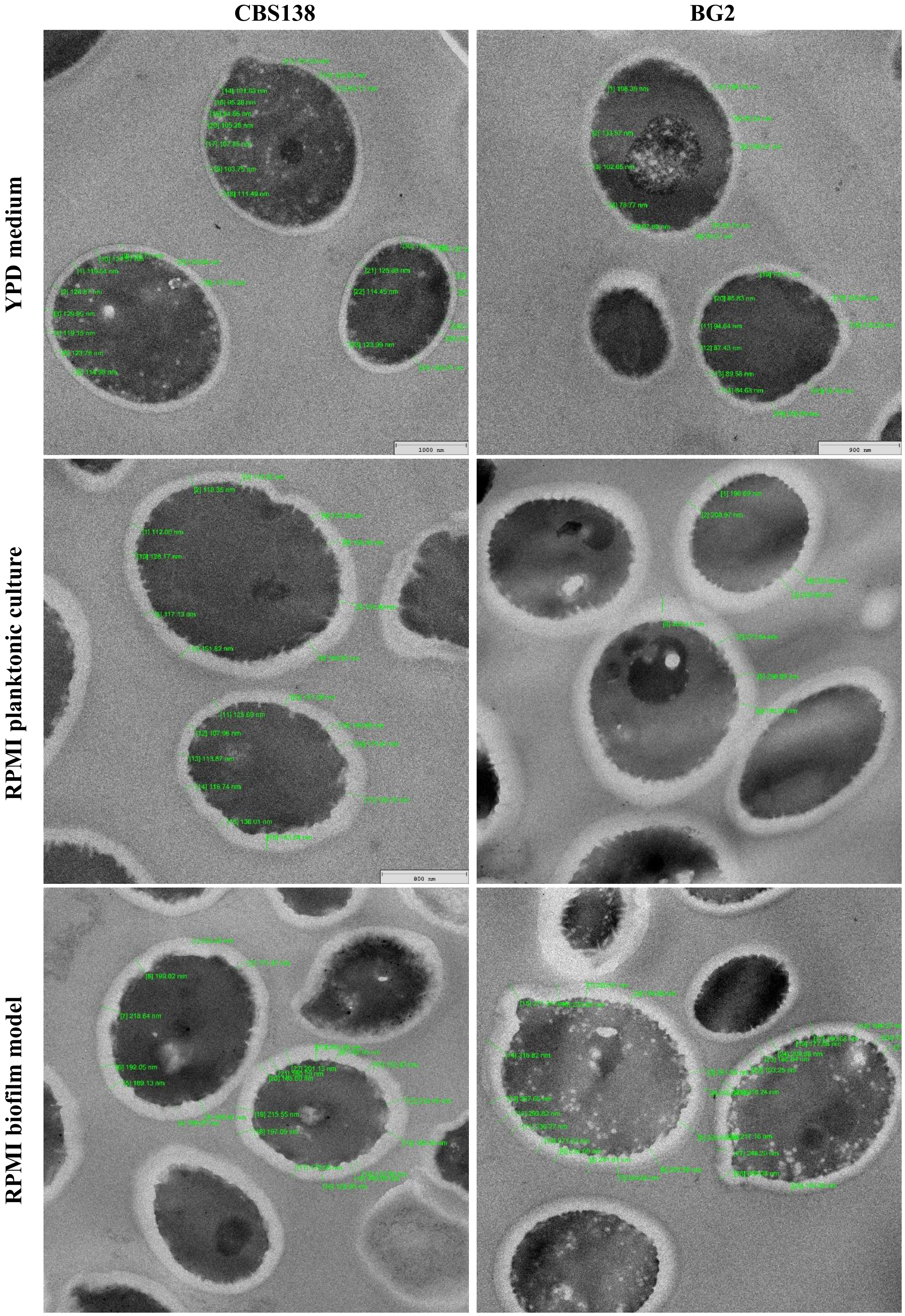

Fig. S1. Example transmission electron microscopy fields of view illustrating the cell wall thickness measurement strategy. Representative transmission electron microscopy (TEM) micrographs of *Candida glabrata* CBS138 and BG2 collected after 24 h under three growth conditions used for surface-shaving experiments: YPD-grown blastospores, RPMI planktonic (free-floating) culture, and the RPMI biofilm model. For each field of view, multiple analysable cells are shown, and total cell wall thickness was quantified by drawing multiple independent transects per cell at distinct positions along the perimeter (green lines). Cell-level measurements were pooled within each biological replicate to generate replicate-level distributions. Scale bars as indicated. The corresponding quantitative comparisons are summarized in Fig. 2B and Supplementary Tab. S1.

Tab. S1. Quantitative measurements of total cell wall thickness by transmission electron microscopy. Numerical values of total cell wall thickness measured from transmission electron microscopy (TEM) micrographs of *Candida glabrata* strains CBS138 and BG2 after 24 hours of growth in YPD culture, planktonic (free-floating) RPMI culture, and RPMI biofilm model. For each biological replicate independent measurements were taken at different locations along the perimeter of each analysed cell within the field of view; cell-level values were combined to generate replicate-level distributions.

**CSB138**			**BG2**		
YPD medium	RPMI planktonic culture	RPMI biofilm model	YPD medium	RPMI planktonic culture	RPMI biofilm model
119.54	143.02	211.46	108.38	198.69	249.39
128.87	146.55	210.12	133.57	208.97	367.35
129.95	140.68	193.48	102.05	200.65	242.49
119.15	186.74	228.42	78.77	203.06	239.99
123.76	177.34	214.27	97.69	256.89	213.06
114.98	179.95	218.67	76.27	198.07	245.27
120.9	186.37	159.17	82.74	273.54	222.29
117.16	141.82	182.5	109.37	262.41	233.95
125.71	152.13	210.1	85.24		245.14
121.57	156.92	179.24	106.16		277.52
101.53	165.47	147.66	94.64		262.41
103.67	171.25	181.35	87.43		241.09
83.17	173.82	161.66	89.58		292.83
101.63	131.16	244.31	84.63		
94.66	169.87	225.41	100.65		
95.28	157.34	212.41	79.74		
107.85	166.67	233.6	123.48		
111.49	141.9	279.81	132.22		
103.75	144.19	217.43	73.71		
105.26	188.12	230.15	85.83		
125.88	112	258.78			
114.45	118.35	235.33			
123.99	112.82	275.04			
133.27	151.82	243.13			
110.72	117.13				
113.07	159.97				
118.77	151.99				
112.12	159.46				
127.36	124.36				
118.04	128.17				
	125.69				
	107.06				
	113.87				
	116.74				
	136.01				
	153.64				
	184.13				
	171.41				
	146.69				
	131.08				

Tab. S2. Cell surface proteins identified in *Candida glabrata* (*Nakaseomyces glabratus*) CBS138 and BG2 strains under different growth conditions, with protein abundance expressed as SAF values. Cell surface shaving of intact fungal cells was performed using trypsin, followed by additional peptide digestion for 24 h. Peptides were analyzed by UHPLC–MS/MS using a Dionex Ultimate 3000 system coupled to an HCTUltra ETDII mass spectrometer. Protein identification was performed by searching MS/MS spectra against the *Candida* (*Nakaseomyces)* NCBI protein database using an in-house Mascot server. Only proteins detected in at least two out of three biological replicates were included.

Accession number	Protein	SAF value (rep.1)	SAF value (rep.2)	SAF value (rep.3)
BG2 blastospores (YPD, 24 h)				
QNG12609.1	Glycosyl hydrolase Utr2; Crh family [*Nakaseomyces glabratus*]	0.00623	0.00415	0.00832
QNG13480.1	1.3-beta-glucanosyltransferase (Gas4) [*Nakaseomyces glabratus*]		0.00573	0.00574
QNG13635.1	GPI-anchored cell wall protein Cwp1.2 [*Nakaseomyces glabratus*]		0.01415	0.01376
QNG14677.1	Cell wall protein Pir4 [*Nakaseomyces glabratus*]		0.01716	0.02146
QNG14678.1	Cell wall protein Pir2 [*Nakaseomyces glabratus*]	0.00623	0.01246	0.01246
QNG15130.1	Asparaginase (Asnase) [*Nakaseomyces glabratus*]	0.00815	0.01902	0.02446
QNG16783.1	Cell wall glycoprotein Ecm33 [*Nakaseomyces glabratus*]	0.00950	0.01188	0.01188
QNG17076.1	Cell wall protein Pir3 [*Nakaseomyces glabratus*]	0.01791	0.02089	0.02687
QNG17077.1	Cell wall protein Pir5 [*Nakaseomyces glabratus*]		0.01905	0.01905
BG2 blastospores (YPD, 48 h)				
QNG12609.1	Glycosyl hydrolase Utr2; Crh family [*Nakaseomyces glabratus*]	0.00415	0.00416	
QNG13237.1	Cell wall glycoprotein Pst1 [*Nakaseomyces glabratus*]	0.00466	0.00466	
QNG13480.1	1.3-beta-glucanosyltransferase (Gas4) [*Nakaseomyces glabratus*]	0.00382	0.00574	0.00574
QNG13635.1	GPI-anchored cell wall protein Cwp1.2 [*Nakaseomyces glabratus*]	0.01415	0.01415	
QNG13716.1	Transglycosidase Scw4 [*Nakaseomyces glabratus*]	0.01329	0.01596	0.00532
QNG14105.1	Glyceraldehyde-3-phosphate dehydrogenase Tdh3 [*Nakaseomyces glabratus*]	0.01506	0.01506	
QNG14677.1	Cell wall protein Pir4 [*Nakaseomyces glabratus*]		0.01288	0.02146
QNG14678.1	Cell wall protein Pir2 [*Nakaseomyces glabratus*]	0.01246	0.00935	0.01246
QNG15130.1	Asparaginase (Asnase) [*Nakaseomyces glabratus*]	0.01358	0.01630	0.01630
QNG16783.1	Cell wall glycoprotein Ecm33 [*Nakaseomyces glabratus*]		0.00951	0.01425
QNG17076.1	Cell wall protein Pir3 [*Nakaseomyces glabratus*]	0.01791	0.02687	0.02089

QNG17077.1	Cell wall protein Pir5 [*Nakaseomyces glabratus*]	0.0095	0.01429	0.01429
CSB 138 blastospores (YPD, 24 h)				
CAG61114.1	60S ribosomal protein L27A (Rpl27a) [*Nakaseomyces glabratus*]	0.02013		0.02685
QNG12167.1	60S ribosomal protein L24e (Rpl24e) [*Nakaseomyces glabratus*]	0.03870		0.02581
QNG12242.1	Elongation factor 2 (Eft2) [*Nakaseomyces glabratus*]		0.00475	0.01188
QNG12244.1	60S ribosomal protein L19e (Rpl19e) [*Nakaseomyces glabratus*]	0.03703		0.02646
QNG12298.1	40S ribosomal protein S8 (Rps8) [*Nakaseomyces glabratus*]	0.02985	0.00995	0.02985
QNG12457.1	Translation elongation factor 3 (Tef3) [*Nakaseomyces glabratus*]	0.00861	0.00669	0.01148
QNG12492.1	60S ribosomal protein L4 (Rpl4) [*Nakaseomyces glabratus*]	0.03038	0.01934	0.03038
QNG12527.1	Cell wall adhesin Epa6 [*Nakaseomyces glabratus*]	0.00419	0.00279	0.00279
QNG12741.1	Heat shock protein Ssb1; Hsp70 family [*Nakaseomyces glabratus*]	0.02446	0.02447	0.02773
QNG12785.1	60S ribosomal protein L31e (Rpl31e) [*Nakaseomyces glabratus*]	0.02654	0.04425	0.04425
QNG12811.1	Translation elongation factor 1-alpha (Tef1) [*Nakaseomyces glabratus*]	0.02620	0.03057	0.03493
QNG13076.1	Uncharacterized Mpt4-like protein [*Nakaseomyces glabratus*]	0.02554	0.01459	0.02189
QNG13127.1	60S ribosomal protein L18 (Rpl18) [*Nakaseomyces glabratus*]	0.01613	0.02150	0.02688
QNG13206.1	U4/U6.U5 small nuclear ribonucleoprotein Snu13 [*Nakaseomyces glabratus*]	0.00781		0.01171
QNG13253.1	60S ribosomal protein L6 (Rpl6) [*Nakaseomyces glabratus*]	0.01047		0.02094
QNG13390.1	Serine hydroxymethyltransferase (Shmt) [*Nakaseomyces glabratus*]	0.00639	0.00639	0.00426
QNG13678.1	Elongation factor 1-beta (Efb1) [*Nakaseomyces glabratus*]	0.00966	0.01932	0.01932
QNG13697.1	40S ribosomal protein S4 (Rps4) [*Nakaseomyces glabratus*]		0.01533	0.01533
QNG13746.1	60S ribosomal protein L26 (Rpl26) [*Nakaseomyces glabratus*]	0.02362		0.03149
QNG13778.1	60S ribosomal protein L11 (Rpl11) [*Nakaseomyces glabratus*]	0.03448	0.03448	0.02874
QNG13790.1	60S ribosomal protein L3 (Rpl3) [*Nakaseomyces glabratus*]	0.02583	0.02326	0.03101
QNG13857.1	60S ribosomal protein L23 (Rpl23) [*Nakaseomyces glabratus*]	0.02189		0.01459
QNG13867.1	Heat shock protein Ssa1; Hsp70 family [*Nakaseomyces glabratus*]	0.00312		0.0125
QNG13979.1	40S ribosomal protein S7e (Rps7e) [*Nakaseomyces glabratus*]	0.03191	0.02659	0.02659
QNG14091.1	60S ribosomal protein L30 (Rpl30) [*Nakaseomyces glabratus*]		0.01229	0.02049
QNG14105.1	Glyceraldehyde-3-phosphate dehydrogenase Tdh3 [*Nakaseomyces glabratus*]	0.04216	0.042169	0.0512
QNG14331.1	60S ribosomal protein L32 (Rpl32) [*Nakaseomyces glabratus*]	0.05343		0.03817
QNG14382.1	40S ribosomal protein S6e (Rps6e) [*Nakaseomyces glabratus*]	0.029661	0.02966	0.02966

QNG14750.1	Alcohol dehydrogenase 1 (Adh1) [*Nakaseomyces glabratus*]	0.01988	0.01705	0.02557
QNG14906.1	40S ribosomal protein S27 (Rps27) [*Nakaseomyces glabratus*]	0.04878	0.02439	0.04878
QNG14969.1	60S ribosomal protein L8 (Rpl8) [*Nakaseomyces glabratus*]	0.03149	0.00787	0.02756
QNG15006.1	40S ribosomal protein S24 (Rps24) [*Nakaseomyces glabratus*]	0.0296	0.01481	
QNG15130.1	Asparaginase (Asnase) [*Nakaseomyces glabratus*]	0.00271	0.01631	0.01902
QNG15266.1	60S ribosomal protein L29 (Rpl29) [*Nakaseomyces glabratus*]	0.03333		0.025
QNG15313.1	60S ribosomal protein L16A (Rpl16a) [*Nakaseomyces glabratus*]		0.02	0.03
QNG15361.1	40S ribosomal protein S3 (Rps3) [*Nakaseomyces glabratus*]		0.02024	0.02024
QNG15418.1	40S ribosomal protein S14 (Rps14) [*Nakaseomyces glabratus*]	0.01449		0.02899
QNG15482.1	40S ribosomal protein S26e (Rps26e) [*Nakaseomyces glabratus*]	0.01680	0.02521	0.01681
QNG15592.1	40S ribosomal protein S15A (Rps15a) [*Nakaseomyces glabratus*]	0.01538	0.02308	0.02308
QNG15714.1	60S ribosomal protein L20 (Rpl20) [*Nakaseomyces glabratus*]	0.04	0.03429	0.04
QNG15862.1	60S ribosomal protein L36e (Rpl36e) [*Nakaseomyces glabratus*]	0.05	0.06	0.06
QNG15898.1	40S ribosomal protein S11B (Rps11b) [*Nakaseomyces glabratus*]	0.01282	0.02564	0.03846
QNG15903.1	Putative flavodoxin; FMN reductase family (Fld1) [*Nakaseomyces glabratus*]	0.03030	0.0303	
QNG15945.1	60S ribosomal protein L10 (Rpl10) [*Nakaseomyces glabratus*]	0.03619		0.02262
QNG16054.1	Enolase 1 (Eno1) [*Nakaseomyces glabratus*]	0.02288	0.01373	0.01602
QNG16314.1	60S ribosomal protein L13e (Rpl13e) [*Nakaseomyces glabratus*]	0.02010	0.01508	0.01508
QNG16402.1	40S ribosomal protein S23 (Rps23) [*Nakaseomyces glabratus*]		0.02759	0.02759
QNG16739.1	40S ribosomal protein S3Ae (Rps3ae) [*Nakaseomyces glabratus*]	0.05882	0.02745	0.02745
QNG16822.1	60S ribosomal protein L5 (Rpl5) [*Nakaseomyces glabratus*]	0.01346	0.01347	0.0101
QNG16993.1	60S ribosomal protein L21 (Rpl21) [*Nakaseomyces glabratus*]	0.03125	0.03125	0.025
QNG17053.1	Pyruvate decarboxylase (Pdc1) [*Nakaseomyces glabratus*]	0.03368	0.02482	0.039
QNG17061.1	60S ribosomal protein L17 (Rpl17) [*Nakaseomyces glabratus*]	0.02717	0.01630	0.01630
QNG17155.1	60S ribosomal protein L14 (Rpl14) [*Nakaseomyces glabratus*]	0.01459	0.02919	0.04379
QNG17234.1	Pyruvate kinase 1 (Pyk1) [*Nakaseomyces glabratus*]		0.00998	0.02395
CSB 138 blastospores (YPD, 48 h)				
CAG57755.1	Uncharacterized Ycl049c-like protein [*Nakaseomyces glabratus*]	0.01470	0.01103	
QNG12167.1	60S ribosomal protein L24e (Rpl24e) [*Nakaseomyces glabratus*]	0.02580	0.03226	0.02581
QNG12242.1	Elongation factor 2 (Eft2) [*Nakaseomyces glabratus*]		0.00713	0.0095

QNG12244.1	60S ribosomal protein L19e (Rpl19e) [*Nakaseomyces glabratus*]	0.02116	0.02646	
QNG12298.1	40S ribosomal protein S8 (Rps8) [*Nakaseomyces glabratus*]	0.02487	0.02488	0.0199
QNG12457.1	Translation elongation factor 3 (Tef3) [*Nakaseomyces glabratus*]		0.01244	0.01148
QNG12492.1	60S ribosomal protein L4 (Rpl4) [*Nakaseomyces glabratus*]	0.02486	0.02209	0.01657
QNG12527.1	Cell wall adhesin Epa6 [*Nakaseomyces glabratus*]	0.00279	0.00419	0.00139
QNG12741.1	Heat shock protein Ssb1; Hsp70 family [*Nakaseomyces glabratus*]	0.02446	0.02121	0.0261
QNG12785.1	60S ribosomal protein L31e (Rpl31e) [*Nakaseomyces glabratus*]	0.03539	0.04425	0.02655
QNG12811.1	Translation elongation factor 1-alpha (Tef1) [*Nakaseomyces glabratus*]	0.03056	0.02183	0.0393
QNG13076.1	Uncharacterized Mpt4-like protein [*Nakaseomyces glabratus*]	0.01824	0.02189	
QNG13126.1	40S ribosomal protein S19e (Rps19e) [*Nakaseomyces glabratus*]	0.02777	0.03472	0.02778
QNG13127.1	60S ribosomal protein L18 (Rpl18) [*Nakaseomyces glabratus*]	0.03225	0.02688	
QNG13206.1	U4/U6.U5 small nuclear ribonucleoprotein Snu13 [*Nakaseomyces glabratus*]	0.01562	0.01953	0.0117
QNG13253.1	60S ribosomal protein L6 (Rpl6) [*Nakaseomyces glabratus*]	0.02617		0.02094
QNG13363.1	40S ribosomal protein S15 (Rps15) [*Nakaseomyces glabratus*]	0.01398	0.02098	
QNG13390.1	Serine hydroxymethyltransferase (Shmt) [*Nakaseomyces glabratus*]	0.00852	0.00853	
QNG13440.1	60S ribosomal protein L12 (Rpl12) [*Nakaseomyces glabratus*]		0.01818	0.01212
QNG13480.1	1.3-beta-glucanosyltransferase (Gas4) [*Nakaseomyces glabratus*]		0.00382	0.00382
QNG13697.1	40S ribosomal protein S4 (Rps4) [*Nakaseomyces glabratus*]		0.01916	0.01916
QNG13742.1	60S acidic ribosomal protein P0 [*Nakaseomyces glabratus*]	0.00643		0.00643
QNG13746.1	60S ribosomal protein L26 (Rpl26) [*Nakaseomyces glabratus*]		0.03149	0.01575
QNG13778.1	60S ribosomal protein L11 (Rpl11) [*Nakaseomyces glabratus*]	0.03448	0.03448	0.01724
QNG13790.1	60S ribosomal protein L3 (Rpl3) [*Nakaseomyces glabratus*]	0.02583	0.03359	0.02326
QNG13867.1	Heat shock protein Ssa1; Hsp70 family [*Nakaseomyces glabratus*]	0.0125	0.00469	
QNG13915.1	40S ribosomal protein S13 (Rps13) [*Nakaseomyces glabratus*]	0.01986		0.01324
QNG13979.1	40S ribosomal protein S7e (Rps7e) [*Nakaseomyces glabratus*]	0.03191	0.03723	0.02659
QNG14105.1	Glyceraldehyde-3-phosphate dehydrogenase Tdh3 [*Nakaseomyces glabratus*]	0.04518	0.0512	0.04217
QNG14152.1	40S ribosomal protein S7B (Rps7b) [*Nakaseomyces glabratus*]	0.01777	0.02667	0.02222
QNG14331.1	60S ribosomal protein L32 (Rpl32) [*Nakaseomyces glabratus*]	0.05343	0.04581	0.0458
QNG14382.1	40S ribosomal protein S6e (Rps6e) [*Nakaseomyces glabratus*]	0.03389	0.02966	0.03389
QNG14750.1	Alcohol dehydrogenase 1 (Adh1) [*Nakaseomyces glabratus*]	0.01704	0.01989	0.01705

QNG14906.1	40S ribosomal protein S27 (Rps27) [*Nakaseomyces glabratus*]	0.03658	0.03659	0.04878
QNG14969.1	60S ribosomal protein L8 (Rpl8) [*Nakaseomyces glabratus*]		0.03543	0.01969
QNG15006.1	40S ribosomal protein S24 (Rps24) [*Nakaseomyces glabratus*]	0.03703	0.01481	0.01481
QNG15130.1	Asparaginase (Asnase) [*Nakaseomyces glabratus*]	0.01358	0.0163	0.01902
QNG15266.1	60S ribosomal protein L29 (Rpl29) [*Nakaseomyces glabratus*]	0.01666	0.025	0.01667
QNG15313.1	60S ribosomal protein L16A (Rpl16a) [*Nakaseomyces glabratus*]	0.04	0.035	0.035
QNG15324.1	60S ribosomal protein L27a [*Nakaseomyces glabratus*]	0.01342	0.01342	0.01342
QNG15361.1	40S ribosomal protein S3 (Rps3) [*Nakaseomyces glabratus*]	0.02024	0.02024	0.02024
QNG15418.1	40S ribosomal protein S14 (Rps14) [*Nakaseomyces glabratus*]	0.02173	0.02899	0.02174
QNG15482.1	40S ribosomal protein S26e (Rps26e) [*Nakaseomyces glabratus*]	0.02521	0.01681	0.02521
QNG15527.1	30S ribosomal protein S10 (Rps10) [*Nakaseomyces glabratus*]	0.02521	0.04201	
QNG15592.1	40S ribosomal protein S15A (Rps15a) [*Nakaseomyces glabratus*]		0.02308	0.01538
QNG15658.1	40S ribosomal protein S17 (Rps17) [*Nakaseomyces glabratus*]	0.02205	0.03676	0.02941
QNG15714.1	60S ribosomal protein L20 (Rpl20) [*Nakaseomyces glabratus*]	0.04571	0.03429	0.03429
QNG15862.1	60S ribosomal protein L36e (Rpl36e) [*Nakaseomyces glabratus*]	0.06	0.05	0.06
QNG15898.1	40S ribosomal protein S11B (Rps11b) [*Nakaseomyces glabratus*]		0.02564	0.02564
QNG15903.1	Putative flavodoxin; FMN reductase family (Fld1) [*Nakaseomyces glabratus*]		0.0202	0.02525
QNG15945.1	60S ribosomal protein L10 (Rpl10) [*Nakaseomyces glabratus*]	0.02262	0.01809	0.02715
QNG15982.1	40S ribosomal protein S16 (Rps16) [*Nakaseomyces glabratus*]	0.02097	0.01399	0.01911
QNG16054.1	Enolase 1 (Eno1) [*Nakaseomyces glabratus*]	0.02745	0.02974	0.02746
QNG16230.1	Fructose-bisphosphate aldolase (Fba1) [*Nakaseomyces glabratus*]		0.00831	0.00831
QNG16314.1	60S ribosomal protein L13e (Rpl13e) [*Nakaseomyces glabratus*]	0.02010	0.01508	
QNG16623.1	60S ribosomal protein L6. KOW motif-containing (Rpl6-KOW) [*Nakaseomyces glabratus*]	0.02840	0.03409	0.03409
QNG16739.1	40S ribosomal protein S3Ae (Rps3ae) [*Nakaseomyces glabratus*]	0.02352	0.03137	0.03529
QNG16822.1	60S ribosomal protein L5 (Rpl5) [*Nakaseomyces glabratus*]	0.03030	0.01684	0.0202
QNG16828.1	40S ribosomal protein SA (Rps2-sa) [*Nakaseomyces glabratus*]	0.01195	0.00797	
QNG16992.1	40S ribosomal protein S9 (Rps9) [*Nakaseomyces glabratus*]	0.02577	0.02577	0.02577
QNG16993.1	60S ribosomal protein L21 (Rpl21) [*Nakaseomyces glabratus*]		0.025	0.025
QNG17053.1	Pyruvate decarboxylase (Pdc1) [*Nakaseomyces glabratus*]	0.03723	0.03369	0.03901
QNG17061.1	60S ribosomal protein L17 (Rpl17) [*Nakaseomyces glabratus*]	0.02173	0.02174	0.03261

QNG17155.1	60S ribosomal protein L14 (Rpl14) [*Nakaseomyces glabratus*]	0.02919	0.0365	0.03649
QNG17234.1	Pyruvate kinase 1 (Pyk1) [*Nakaseomyces glabratus*]		0.00798	0.00798
CSB 138 free-floating aggregates (RPMI, 24 h)				
QNG12527.1	Cell wall adhesin Epa6 [*Nakaseomyces glabratus*]		0.00839	0.00979
QNG12811.1	Translation elongation factor 1-alpha (Tef1) [*Nakaseomyces glabratus*]	0.01310	0.02402	0.01528
QNG13480.1	1.3-beta-glucanosyltransferase (Gas4) [*Nakaseomyces glabratus*]	0.00573	0.00574	0.00382
QNG13636.1	GPI-anchored cell wall protein Cwp1.1 [*Nakaseomyces glabratus*]	0.00917	0.00917	0.00917
QNG13716.1	Transglycosidase Scw4 [*Nakaseomyces glabratus*]	0.01329	0.00798	0.01064
QNG14105.1	Glyceraldehyde-3-phosphate dehydrogenase Tdh3 [*Nakaseomyces glabratus*]	0.02108	0.03614	0.04518
QNG15130.1	Asparaginase (Asnase) [*Nakaseomyces glabratus*]	0.02173	0.02174	0.02717
QNG15396.1	Cell wall adhesin Awp2 [*Nakaseomyces glabratus*]	0.00857	0.00613	0.00368
QNG15616.1	Alpha-trehalase 1 (Ath1) [*Nakaseomyces glabratus*]		0.01320	0.01155
QNG15968.1	Beta-glucosidase (BglC) [*Nakaseomyces glabratus*]	0.01354	0.00451	
QNG16054.1	Enolase 1 (Eno1) [*Nakaseomyces glabratus*]	0.01144	0.01831	0.03432
QNG16783.1	Cell wall glycoprotein Ecm33 [*Nakaseomyces glabratus*]	0.00475		0.00950
QNG17053.1	Pyruvate decarboxylase (Pdc1) [*Nakaseomyces glabratus*]		0.02128	0.01596
CSB 138 free-floating aggregates (RPMI, 48 h)				
CAG62903.1	60S ribosomal protein L38e (Rpl38e) [*Nakaseomyces glabratus*]	0.01226		0.01227
QNG12527.1	Cell wall adhesin Epa6 [*Nakaseomyces glabratus*]	0.00419		0.00559
QNG12811.1	Translation elongation factor 1-alpha (Tef1) [*Nakaseomyces glabratus*]	0.01746		0.01528
QNG13716.1	Transglycosidase Scw4 [*Nakaseomyces glabratus*]	0.01063		0.00798
QNG14105.1	Glyceraldehyde-3-phosphate dehydrogenase Tdh3 [*Nakaseomyces glabratus*]	0.04819	0.04819	0.04518
QNG15049.1	Heat shock protein 12 (Hsp12) [*Nakaseomyces glabratus*]		0.03883	0.08738
QNG15130.1	Asparaginase (Asnase) [*Nakaseomyces glabratus*]	0.02445	0.01630	0.02989
QNG16054.1	Enolase 1 (Eno1) [*Nakaseomyces glabratus*]	0.04118	0.03204	0.04348
QNG17053.1	Pyruvate decarboxylase (Pdc1) [*Nakaseomyces glabratus*]	0.02836	0.01418	0.02837
BG2 free-floating aggregates (RPMI, 24 h)				
QNG12440.1	Transaldolase 1 (Tal1) [*Nakaseomyces glabratus*]		0.00898	0.008982036
QNG12811.1	Translation elongation factor 1-alpha (Tef1) [*Nakaseomyces glabratus*]	0.017467	0.01747	0.032751092
QNG13294.1	Hydrophilin Gre1 [*Nakaseomyces glabratus*]		0.03521	0.028169014

QNG13311.1	Phosphoglycerate mutase (Gpm1) [*Nakaseomyces glabratus*]		0.02834	0.02024
QNG13716.1	Transglycosidase Scw4 [*Nakaseomyces glabratus*]	0.01595	0.01064	0.00532
QNG14105.1	Glyceraldehyde-3-phosphate dehydrogenase Tdh3 [*Nakaseomyces glabratus*]	0.03012	0.04217	0.05422
QNG14615.1	Acetyl-CoA synthetase (Acs1) [*Nakaseomyces glabratus*]		0.00994	0.0071
QNG14998.1	Triosephosphate isomerase-like protein (Tim-like) [*Nakaseomyces glabratus*]		0.00723	0.00723
QNG15049.1	Heat shock protein 12 (Hsp12) [*Nakaseomyces glabratus*]	0.09708	0.12621	0.09709
QNG15130.1	Asparaginase (Asnase) [*Nakaseomyces glabratus*]		0.01630	0.0163
QNG15376.1	Uncharacterized Ynl195c-like mitochondrial protein [*Nakaseomyces glabratus*]		0.00765	0.00765
QNG16054.1	Enolase 1 (Eno1) [*Nakaseomyces glabratus*]	0.03203	0.04119	0.04119
QNG16230.1	Fructose-bisphosphate aldolase (Fba1) [*Nakaseomyces glabratus*]		0.01939	0.01662
QNG16783.1	Cell wall glycoprotein Ecm33 [*Nakaseomyces glabratus*]	0.00475		0.00475
QNG17053.1	Pyruvate decarboxylase (Pdc1) [*Nakaseomyces glabratus*]	0.01595	0.0195	0.02128
QNG17076.1	Cell wall protein Pir3 [*Nakaseomyces glabratus*]	0.00895	0.01194	0.01493
QNG17234.1	Pyruvate kinase 1 (Pyk1) [*Nakaseomyces glabratus*]		0.01997	0.01996
QNG17290.1	6-phosphogluconate dehydrogenase Gnd1 [*Nakaseomyces glabratus*]		0.00818	0.00818
BG2 free-floating aggregates (RPMI, 48 h)				
QNG12440.1	Transaldolase 1 (Tal1) [*Nakaseomyces glabratus*]	0.0059		0.01796
QNG12811.1	Translation elongation factor 1-alpha (Tef1) [*Nakaseomyces glabratus*]	0.02183	0.02621	0.02402
QNG13294.1	Hydrophilin Gre1 [*Nakaseomyces glabratus*]		0.02817	0.04225
QNG13311.1	Phosphoglycerate mutase (Gpm1) [*Nakaseomyces glabratus*]		0.02429	0.02834
QNG14105.1	Glyceraldehyde-3-phosphate dehydrogenase Tdh3 [*Nakaseomyces glabratus*]	0.05120	0.05723	0.0512
QNG14750.1	Alcohol dehydrogenase 1 (Adh1) [*Nakaseomyces glabratus*]	0.00852	0.0142	0.01705
QNG14998.1	Triosephosphate isomerase-like protein (Tim-like) [*Nakaseomyces glabratus*]		0.01266	0.01627
QNG15049.1	Heat shock protein 12 (Hsp12) [*Nakaseomyces glabratus*]	0.09708	0.13592	0.12621
QNG15130.1	Asparaginase (Asnase) [*Nakaseomyces glabratus*]	0.00815	0.00815	0.01359
QNG16054.1	Enolase 1 (Eno1) [*Nakaseomyces glabratus*]	0.03661	0.04577	0.0481
QNG16230.1	Fructose-bisphosphate aldolase (Fba1) [*Nakaseomyces glabratus*]	0.01939	0.01108	
QNG17053.1	Pyruvate decarboxylase (Pdc1) [*Nakaseomyces glabratus*]	0.02304	0.03014	0.02659
QNG17234.1	Pyruvate kinase 1 (Pyk1) [*Nakaseomyces glabratus*]	0.00798	0.00998	0.02395
QNG17290.1	6-phosphogluconate dehydrogenase Gnd1 [*Nakaseomyces glabratus*]		0.00818	0.01022

CSB 138 biofilm (RPMI, 24 h)				
CAG61765.1	Heat shock protein 90 (Hsp90) [*Nakaseomyces glabratus*]	0.00851		0.01418
QNG12242.1	Elongation factor 2 (Eft2) [*Nakaseomyces glabratus*]	0.01068	0.00594	0.00713
QNG12440.1	Transaldolase 1 (Tal1) [*Nakaseomyces glabratus*]	0.02395	0.02695	
QNG12527.1	Cell wall adhesin Epa6 [*Nakaseomyces glabratus*]	0.00279	0.00279	0.00279
QNG12741.1	Heat shock protein Ssb1; Hsp70 family [*Nakaseomyces glabratus*]	0.00652	0.00816	0.00653
QNG12811.1	Translation elongation factor 1-alpha (Tef1) [*Nakaseomyces glabratus*]	0.02838	0.02838	0.02838
QNG12874.1	Endoglucanase Egt2 [*Nakaseomyces glabratus*]	0.00296	0.00296	
QNG13114.1	GPI-anchored aspartic protease Yps3 [*Nakaseomyces glabratus*]	0.01113	0.01299	0.01113
QNG13311.1	Phosphoglycerate mutase (Gpm1) [*Nakaseomyces glabratus*]	0.01214	0.02429	0.01215
QNG13345.1	Glucokinase (Glk1) [*Nakaseomyces glabratus*]	0.00606	0.0101	0.00404
QNG13716.1	Transglycosidase Scw4 [*Nakaseomyces glabratus*]	0.02659	0.03191	0.02926
QNG13844.1	Heat shock protein Ssa3; Hsp70 family [*Nakaseomyces glabratus*]	0.00618	0.00464	0.00464
QNG13867.1	Heat shock protein Ssa1; Hsp70 family [*Nakaseomyces glabratus*]	0.00937	0.01093	0.01094
QNG14105.1	Glyceraldehyde-3-phosphate dehydrogenase Tdh3 [*Nakaseomyces glabratus*]	0.05722	0.0512	0.05121
QNG14505.1	Triosephosphate isomerase (Tpi1) [*Nakaseomyces glabratus*]		0.00806	0.00806
QNG14572.1	Inorganic pyrophosphatase (Ipp1) [*Nakaseomyces glabratus*]		0.00697	0.00697
QNG14678.1	Cell wall protein Pir2 [*Nakaseomyces glabratus*]		0.0062	0.00935
QNG15049.1	Heat shock protein 12 (Hsp12) [*Nakaseomyces glabratus*]	0.07766	0.10679	0.09709
QNG15130.1	Asparaginase (Asnase) [*Nakaseomyces glabratus*]	0.02173	0.02446	0.01902
QNG15885.1	Putative adenylate kinase (Adk) [*Nakaseomyces glabratus*]	0.01351	0.00901	
QNG15903.1	Putative flavodoxin; FMN reductase family (Fld1) [*Nakaseomyces glabratus*]	0.02020	0.0101	0.01515
QNG16054.1	Enolase 1 (Eno1) [*Nakaseomyces glabratus*]	0.04347	0.04577	0.03433
QNG16230.1	Fructose-bisphosphate aldolase (Fba1) [*Nakaseomyces glabratus*]	0.01385		0.00831
QNG16278.1	Phosphoglycerate kinase (Pgk1) [*Nakaseomyces glabratus*]	0.00961	0.00721	0.00962
QNG16474.1	Cell wall protein Tos1 [*Nakaseomyces glabratus*]	0.00445		0.00445
QNG16992.1	40S ribosomal protein S9 (Rps9) [*Nakaseomyces glabratus*]	0.01030	0.01031	
QNG17053.1	Pyruvate decarboxylase (Pdc1) [*Nakaseomyces glabratus*]	0.01773	0.0195	0.01773
QNG17076.1	Cell wall protein Pir3 [*Nakaseomyces glabratus*]	0.01194		0.00896
QNG17234.1	Pyruvate kinase 1 (Pyk1) [*Nakaseomyces glabratus*]	0.00998		0.00998

QNG17290.1	6-phosphogluconate dehydrogenase Gnd1 [*Nakaseomyces glabratus*]	0.01022	0.01841	0.01432
CSB 138 biofilm (RPMI, 48 h)				
QNG12242.1	Elongation factor 2 (Eft2) [*Nakaseomyces glabratus*]	0.00831	0.00831	0.0095
QNG12440.1	Transaldolase 1 (Tal1) [*Nakaseomyces glabratus*]	0.02395	0.02395	0.02994
QNG12741.1	Heat shock protein Ssb1; Hsp70 family [*Nakaseomyces glabratus*]	0.00489	0.00653	
QNG12766.1	Heat shock protein Hsc82; Hsp90 family [*Nakaseomyces glabratus*]	0.00709	0.0156	0.0156
QNG12811.1	Translation elongation factor 1-alpha (Tef1) [*Nakaseomyces glabratus*]	0.01528	0.01528	0.01747
QNG12817.1	Transketolase 1 (Tkl1) [*Nakaseomyces glabratus*]	0.00294	0.00441	0.00442
QNG13114.1	GPI-anchored aspartic protease Yps3 [*Nakaseomyces glabratus*]	0.02782	0.03339	0.03525
QNG13311.1	Phosphoglycerate mutase (Gpm1) [*Nakaseomyces glabratus*]	0.02024	0.01619	
QNG13716.1	Transglycosidase Scw4 [*Nakaseomyces glabratus*]	0.05053	0.04787	0.04521
QNG13867.1	Heat shock protein Ssa1; Hsp70 family [*Nakaseomyces glabratus*]	0.01093	0.01093	0.00781
QNG14105.1	Glyceraldehyde-3-phosphate dehydrogenase Tdh3 [*Nakaseomyces glabratus*]	0.06024	0.06325	0.06626
QNG14677.1	Cell wall protein Pir4 [*Nakaseomyces glabratus*]	0.01287		0.01717
QNG14678.1	Cell wall protein Pir2 [*Nakaseomyces glabratus*]	0.01869	0.01246	
QNG14750.1	Alcohol dehydrogenase 1 (Adh1) [*Nakaseomyces glabratus*]	0.00284	0.00568	0.00568
QNG15049.1	Heat shock protein 12 (Hsp12) [*Nakaseomyces glabratus*]	0.03883	0.02913	0.01942
QNG15130.1	Asparaginase (Asnase) [*Nakaseomyces glabratus*]	0.02717	0.02717	0.02989
QNG15616.1	Alpha-trehalase 1 (Ath1) [*Nakaseomyces glabratus*]	0.00577	0.00413	0.00743
QNG16054.1	Enolase 1 (Eno1) [*Nakaseomyces glabratus*]	0.04805	0.05492	0.05492
QNG16230.1	Fructose-bisphosphate aldolase (Fba1) [*Nakaseomyces glabratus*]	0.01108	0.01108	0.01385
QNG16278.1	Phosphoglycerate kinase (Pgk1) [*Nakaseomyces glabratus*]	0.01682	0.02404	0.01683
QNG17053.1	Pyruvate decarboxylase (Pdc1) [*Nakaseomyces glabratus*]	0.02127	0.01418	0.02482
QNG17290.1	6-phosphogluconate dehydrogenase Gnd1 [*Nakaseomyces glabratus*]	0.01431	0.01636	
BG2 biofilm (RPMI, 24 h)				
QNG12811.1	Translation elongation factor 1-alpha (Tef1) [*Nakaseomyces glabratus*]		0.01092	0.00873
QNG13237.1	Cell wall glycoprotein Pst1 [*Nakaseomyces glabratus*]	0.00699	0.01166	0.00932
QNG13480.1	1.3-beta-glucanosyltransferase (Gas4) [*Nakaseomyces glabratus*]		0.00382	0.00574
QNG13716.1	Transglycosidase Scw4 [*Nakaseomyces glabratus*]	0.03191	0.0239	0.03723
QNG14105.1	Glyceraldehyde-3-phosphate dehydrogenase Tdh3 [*Nakaseomyces glabratus*]	0.02710	0.01807	0.02108

QNG14677.1	Cell wall protein Pir4 [*Nakaseomyces glabratus*]		0.01717	0.01717
QNG14678.1	Cell wall protein Pir2 [*Nakaseomyces glabratus*]	0.01557	0.00935	0.00935
QNG15049.1	Heat shock protein 12 (Hsp12) [*Nakaseomyces glabratus*]	0.03883		0.05825
QNG15130.1	Asparaginase (Asnase) [*Nakaseomyces glabratus*]	0.02173	0.01359	0.01630
QNG16054.1	Enolase 1 (Eno1) [*Nakaseomyces glabratus*]	0.02288	0.01831	0.00915
QNG16783.1	Cell wall glycoprotein Ecm33 [*Nakaseomyces glabratus*]	0.01901	0.01663	0.02138
BG2 biofilm (RPMI, 48 h)				
QNG12609.1	Glycosyl hydrolase Utr2; Crh family [*Nakaseomyces glabratus*]	0.00623	0.00624	0.00624
QNG12811.1	Translation elongation factor 1-alpha (Tef1) [*Nakaseomyces glabratus*]	0.01091		0.00655
QNG12874.1	Endoglucanase Egt2 [*Nakaseomyces glabratus*]	0.00296	0.00593	0.00741
QNG13114.1	GPI-anchored aspartic protease Yps3 [*Nakaseomyces glabratus*]	0.02411	0.01299	0.02226
QNG13237.1	Cell wall glycoprotein Pst1 [*Nakaseomyces glabratus*]	0.01165		0.00932
QNG13635.1	GPI-anchored cell wall protein Cwp1.2 [*Nakaseomyces glabratus*]	0.02358		0.00943
QNG13711.1	Glycosyl hydrolase family 17 protein (Gh17) [*Nakaseomyces glabratus*]	0.00649	0.00975	0.00974
QNG13716.1	Transglycosidase Scw4 [*Nakaseomyces glabratus*]	0.07446	0.06383	0.07181
QNG14677.1	Cell wall protein Pir4 [*Nakaseomyces glabratus*]	0.02145	0.02146	0.01717
QNG14678.1	Cell wall protein Pir2 [*Nakaseomyces glabratus*]	0.01246	0.01246	0.01246
QNG15130.1	Asparaginase (Asnase) [*Nakaseomyces glabratus*]	0.02989	0.02174	0.02174
QNG15303.1	Beta-glucosidase; SUN family (Sun-like) [*Nakaseomyces glabratus*]	0.01171	0.01874	0.01639
QNG15616.1	Alpha-trehalase 1 (Ath1) [*Nakaseomyces glabratus*]	0.00661		0.01073
QNG15968.1	Beta-glucosidase (BglC) [*Nakaseomyces glabratus*]	0.04514	0.04515	0.0474
QNG16379.1	Beta-glucosidase Sun4 [*Nakaseomyces glabratus*]	0.01159		0.01449
QNG16783.1	Cell wall glycoprotein Ecm33 [*Nakaseomyces glabratus*]	0.02137	0.019	0.01663
QNG16951.1	Cell wall protein Tos1 [*Nakaseomyces glabratus*]	0.01559	0.01114	
QNG17076.1	Cell wall protein Pir3 [*Nakaseomyces glabratus*]	0.01194	0.01791	0.02089
QNG17077.1	Cell wall protein Pir5 [*Nakaseomyces glabratus*]	0.0095	0.00952	0.00952
QNG17133.1	Endochitinase Cts1 [*Nakaseomyces glabratus*]	0.00581	0.00774	0.00774
QNG17311.1	Cell wall mannoprotein Mp65 [*Nakaseomyces glabratus*]	0.02695	0.01887	0.01078

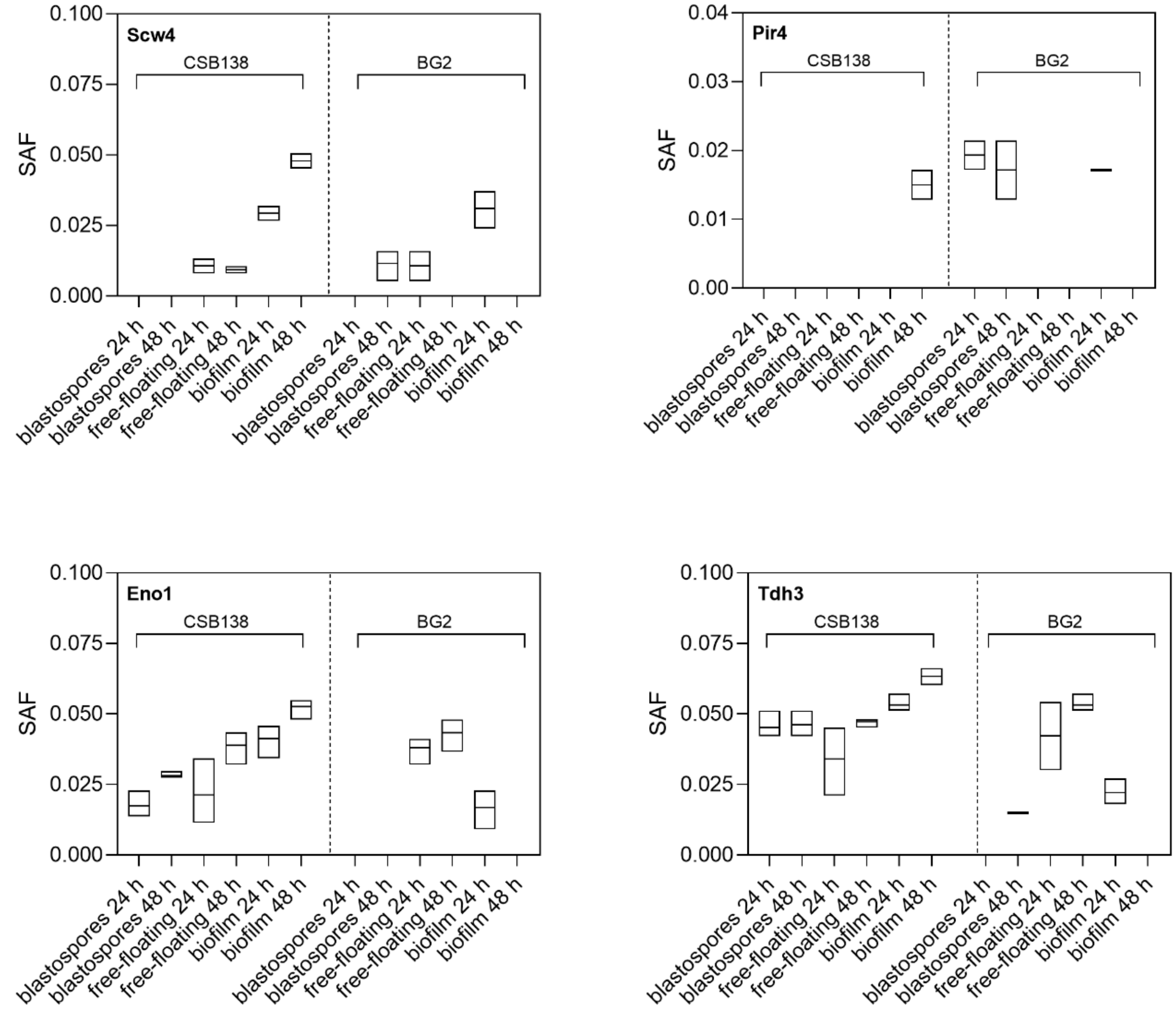

Fig. S2. SAF-based comparison of selected surface-associated proteins in *C. glabrata* CBS138 and BG2. Boxplots show SAF values for two classical cell-wall proteins (Scw4 and Pir4) and two moonlighting proteins (Eno1 and Tdh3) across the indicated growth conditions in both strains. These proteins were selected because of they presence in multiple growth models, enabling comparison of condition-dependent variation in relative surface abundance. Individual values are shown together with the mean and interquartile range. Detailed statistical comparison is presented in Tab. S3.

Tab. S3. Statistical significance of SAF-based comparison of selected surface-associated proteins in *C. glabrata* CBS138 and BG2. Statistical significance was assessed using one way ANOVA with Tukey's multiple comparison test using GraphPad Prism software. The table lists adjusted p values only for comparisons that reached statistical significance.

Culture Conditions	Adjusted P Value
**Scw4**	
free-floating 24 h vs. biofilm 24 h	0.0031
free-floating 24 h vs. biofilm 48 h	<0.0001
free-floating 24 h vs. biofilm 24 h	0.0013
free-floating 24 h vs. biofilm 48 h	<0.0001
free-floating 48 h vs. biofilm 24 h	0.0046
free-floating 48 h vs. biofilm 48 h	<0.0001
free-floating 48 h vs. biofilm 24 h	0.0021
free-floating 48 h vs. biofilm 48 h	<0.0001
biofilm 24 h vs. biofilm 48 h	0.0031
biofilm 24 h vs. blastospores 48 h	0.0049
biofilm 24 h vs. free-floating 24 h	0.0031
biofilm 24 h vs. biofilm 48 h	<0.0001
biofilm 48 h vs. blastospores 48 h	<0.0001
biofilm 48 h vs. free-floating 24 h	<0.0001
biofilm 48 h vs. biofilm 24 h	0.0076
biofilm 48 h vs. biofilm 48 h	0.0006
blastospores 48 h vs. biofilm 24 h	0.002
blastospores 48 h vs. biofilm 48 h	<0.0001
free-floating 24 h vs. biofilm 24 h	0.0013
free-floating 24 h vs. biofilm 48 h	<0.0001
biofilm 24 h vs. biofilm 48 h	<0.0001
**Eno1**	
blastospores 24 h vs. free-floating 48 h	0.0176
blastospores 24 h vs. biofilm 24 h	0.0071
blastospores 24 h vs. biofilm 48 h	<0.0001
blastospores 24 h vs. free-floating 24 h	0.0237
blastospores 24 h vs. free-floating 48 h	0.0029
blastospores 48 h vs. biofilm 48 h	0.0053
blastospores 48 h vs. biofilm 48 h	0.0303
free-floating 24 h vs. biofilm 24 h	0.0318
free-floating 24 h vs. biofilm 48 h	0.0004
free-floating 24 h vs. free-floating 48 h	0.013
free-floating 48 h vs. biofilm 24 h	0.013
free-floating 48 h vs. biofilm 48 h	0.0016
biofilm 24 h vs. biofilm 24 h	0.0053
biofilm 24 h vs. biofilm 48 h	0.0008
biofilm 48 h vs. biofilm 24 h	<0.0001
biofilm 48 h vs. biofilm 48 h	<0.0001
free-floating 24 h vs. biofilm 24 h	0.0176

free-floating 24 h vs. biofilm 48 h	0.0019
free-floating 48 h vs. biofilm 24 h	0.0021
free-floating 48 h vs. biofilm 48 h	0.0004
**Tdh3**	
blastospores 24 h vs. blastospores 48 h	0.0024
blastospores 24 h vs. biofilm 24 h	0.0112
blastospores 24 h vs. biofilm 48 h	0.0003
blastospores 48 h vs. blastospores 48 h	0.0017
blastospores 48 h vs. biofilm 24 h	0.0075
blastospores 48 h vs. biofilm 48 h	0.0003
free-floating 24 h vs. biofilm 48 h	0.001
free-floating 24 h vs. biofilm 48 h	0.0074
free-floating 48 h vs. blastospores 48 h	0.0012
free-floating 48 h vs. biofilm 24 h	0.005
free-floating 48 h vs. biofilm 48 h	0.0002
biofilm 24 h vs. blastospores 48 h	0.0001
biofilm 24 h vs. biofilm 24 h	0.0005
biofilm 24 h vs. biofilm 48 h	<0.0001
biofilm 48 h vs. blastospores 48 h	<0.0001
biofilm 48 h vs. free-floating 24 h	0.0247
biofilm 48 h vs. biofilm 24 h	<0.0001
biofilm 48 h vs. biofilm 48 h	<0.0001
blastospores 48 h vs. free-floating 24 h	0.0071
blastospores 48 h vs. free-floating 48 h	0.0001
free-floating 24 h vs. biofilm 24 h	0.0363
free-floating 24 h vs. biofilm 48 h	0.0008
free-floating 48 h vs. biofilm 24 h	0.0005
free-floating 48 h vs. biofilm 48 h	<0.0001
